# MicroRNAs as Potential Biomarkers for the Diagnosis of Chronic Kidney Disease: A Systematic Review and Meta-Analysis

**DOI:** 10.3389/fmed.2021.782561

**Published:** 2022-02-07

**Authors:** Jing Li, Leilei Ma, Hangxing Yu, Yahong Yao, Zhiyuan Xu, Wei Lin, Lin Wang, Xuejun Wang, Hongtao Yang

**Affiliations:** First Teaching Hospital of Tianjin University of Traditional Chinese Medicine, National Clinical Research Center for Chinese Medicine Acupuncture and Moxibustion, Tianjin, China

**Keywords:** miRNA, CKD, diagnosis, biomarkers, meta-analysis

## Abstract

For Chronic Kidney Disease (CKD), the study of microRNA as a biomarker has become an exciting area, so we carried out a meta-analysis to investigate the potential diagnostic values of miRNAs in CKD. We searched Pubmed, Cochrane Library, Embase, and Web of science databases to identify relevant publications published from the establishment of the database to April 30, 2021. We included a total of 26 articles containing 56 studies. There were 4,098 patients with CKD and 2,450 patients without CKD. We found that the overall sensitivity and specificity of miRNAs in CKD diagnosis were 0.86 (95% CI: 0.83–0.89) and 0.79 (95% CI: 0.75–0.83), respectively. In addition, we plotted the summary receiver operator characteristic (SROC) curve to assess diagnostic accuracy, with the area under the curve (AUC) of 0.90 (95% CI: 0.87–0.92). Subgroup analysis showed that sensitivity, specificity, and AUC of miRNAs in plasma and serum were 0.84, 0.78, 0.88; and 0.79, 0.76, 0.83, respectively, while miRNAs in urine were 0.89 for sensitivity, 0.82 for specificity, and 0.92 for AUC. Moreover, we found that the panel of microRNAs (miRNAs) could improve the pooled sensitivity (0.88, 0.81, and 0.91 for sensitivity, specificity, and AUC, respectively). We believe that miRNAs have great potential to become an effective diagnostic biomarker for CKD. Panels of miRNA have higher accuracy than single miRNAs. Additionally, miRNAs in both blood and urine have significant accuracy in the diagnosis of CKD; nevertheless, urine is superior.

## Introduction

Chronic kidney disease (CKD) refers to a progressive loss of kidney function with kidney damage or glomerular filtration rate (eGFR) <60 ml/min per 1.73 m^2^ for more than 3 months or longer. Renal damage refers to pathological abnormalities or abnormal urine sediment or increased urinary albumin excretion rate detected by imaging or renal biopsy (1). Studies from the United States, Australia, Europe, and other developed countries show that the incidence of CKD is roughly 10–15% in the average individuals (2–4). By 2040, it is expected to be the fifth most widespread cause of death worldwide (5). The CKD appears to be a major global disease that cannot be ignored. Its high incidence, high overhead, high insidiousness, poor prognosis, and other problems seriously endanger the health of people (6).

Most patients with CKD do not have obvious clinical symptoms in the early stage of the disease, so it is difficult to diagnose early. With the further progress of the disease, it may develop into an end-stage renal disease, which can be irreversible. At present, CKD is mainly diagnosed by kidney biopsy, ultrasound imaging, and biological markers (urea nitrogen, creatinine, albuminuria, and nuclide, etc.) detection. Due to traumatic and radioactive problems, renal biopsy and the radionuclide method are difficult to be routinely used in clinics; besides, ultrasound imaging and creatinine are limited in the judgment of renal injury because of their low sensitivity (7). Therefore, the search for biomarkers and methods with high diagnostic efficiency to improve the diagnostic efficiency of CKD has been the focus of clinical appeal.

For CKD, the study of microRNAs (miRNAs/miRs) as biomarkers has become an exciting field. MicroRNA is a class of small non-coding RNA with a length of 20–25 nucleotides; it is estimated that miRNAs can target about 60% of human genes and, therefore, play a very important and extensive role in gene regulation (8, 9). Previous findings suggest that miRNAs have key regulatory actions in the upgrowth, structure, and function of the kidney, including the maintenance of fluid, electrolyte, acid base, and blood pressure. They are also involved in pathological processes. Furthermore, miRNAs are relatively stable in serum and urine regardless of storage condition (10). MiRNAs are undoubtedly valuable as diagnostic and monitoring markers for CKD; as a result, the meta-analysis was performed to investigate the potential diagnostic efficacy of miRNAs in CKD, in the hope of finding a new non-invasive biomarker for CKD diagnosis.

## Materials and Methods

### Search Strategy

Pubmed, Cochrane Library, Embase, and Web of Science were retrieved to search all the literature on the diagnostic value of miRNAs in CKD, which are published from the establishment of the database to April 30. The search formula usually includes three keywords: “kidney disease,” “microRNAs,” and “diagnosis.” (Prospero Registration Number is CRD42021253868,). Specific search strategies such as those in Pubmed are attached at the Supplementary Material S4.

### Inclusion and Exclusion Criteria

Pieces of literature were admitted if all these criteria were met: (i) all patients with CKD were diagnosed by renal biopsy or biomarker examination; (ii) pieces of literature on miRNAs related to CKD diagnosis, which were written in English; (iii) the pieces of literature studied the expression of miRNA in tissues such as urine or blood, and were designed as case-control studies; and (iv) sensitivity, specificity, or true positive (TP), false positive (FP), false negative (FN), true negative (TN) could be provided.

Studies will be excluded if either of the following criteria is met: (i) the full text cannot be obtained or the data are incomplete; (ii) animal experiments; (iii) basic research, reviews, cases reports, letters, reviews, conference abstracts, etc.; (iv) repeated publication.

### Quality Assessment

The Quality Assessment of Diagnostic Accuracy Studies-2 (QUADAS-2) criteria in Revman 5.3 is used to perform an evaluation of the pieces of literature, which consists of four sections: patient selection, index test, reference standard, and flow and timing. The bias risk level can be determined as “low,” “high,” or “unclear” based on the “yes,” “no,” or “unclear” answers to the questions corresponding to each section. If the answers to all the signature questions within a range are all “yes,” they can be assessed as low; If one of the answers is “no,” then it is qualified as “high.” If both are “unclear,” they are judged as “unclear.”

### Data Extraction

Two authors conducted an independent data extraction from the included studies. Data, such as author, headline, publication year, country, study design, disease, experimental group/control group, sample size, eligibility criteria, miRNAs, method of detection, source of tissue, sensitivity, specificity, TP, FP, FN, TN, etc., were extracted.

### Statistical Analysis

Stata 14.0 was used for carrying out all the calculations. We extracted TP, FP, FN, TN, and other diagnostic information to analyze sensitivity (Sens), specificity (Sepc), positive likelihood ratio (PLR), negative likelihood ratio (NLR), diagnostic odds ratio (DOR), and its corresponding *I*^2^ and 95% CI. The summary receiver operator characteristic (SROC) curve also plotted and calculated the area under the curve (AUC) to test the pooled diagnostic value of miRNAs. We analyzed the publication bias with the help of funnel plots, and publication bias was considered to exist between the studies when the *p*-value was <0.05. Heterogeneity was assessed by chi-square and *I*^2^ tests. When a *p*-value <0.01 or *I*^2^ > 50%, heterogeneity is of significance, we would use a random effects model to combine the statistics. Conversely, we would use a fixed effects model. The source of heterogeneity was explored by subgroup analysis, regression analysis, and sensitivity analysis. For the included pieces of literature, we planned to adopt the following classification criteria to explore a potential source of heterogeneity: a. ethnicity; b. profile of miRNAs—single miRNA vs. panel of miRNAs; c. detection method; d. sample size—≥100 vs. <100; e. tissue—serum vs. plasma vs. urine; and f. disease profile.

## Results

### Search Results and Basic Features of Pieces of Literature

**Figure 1:**
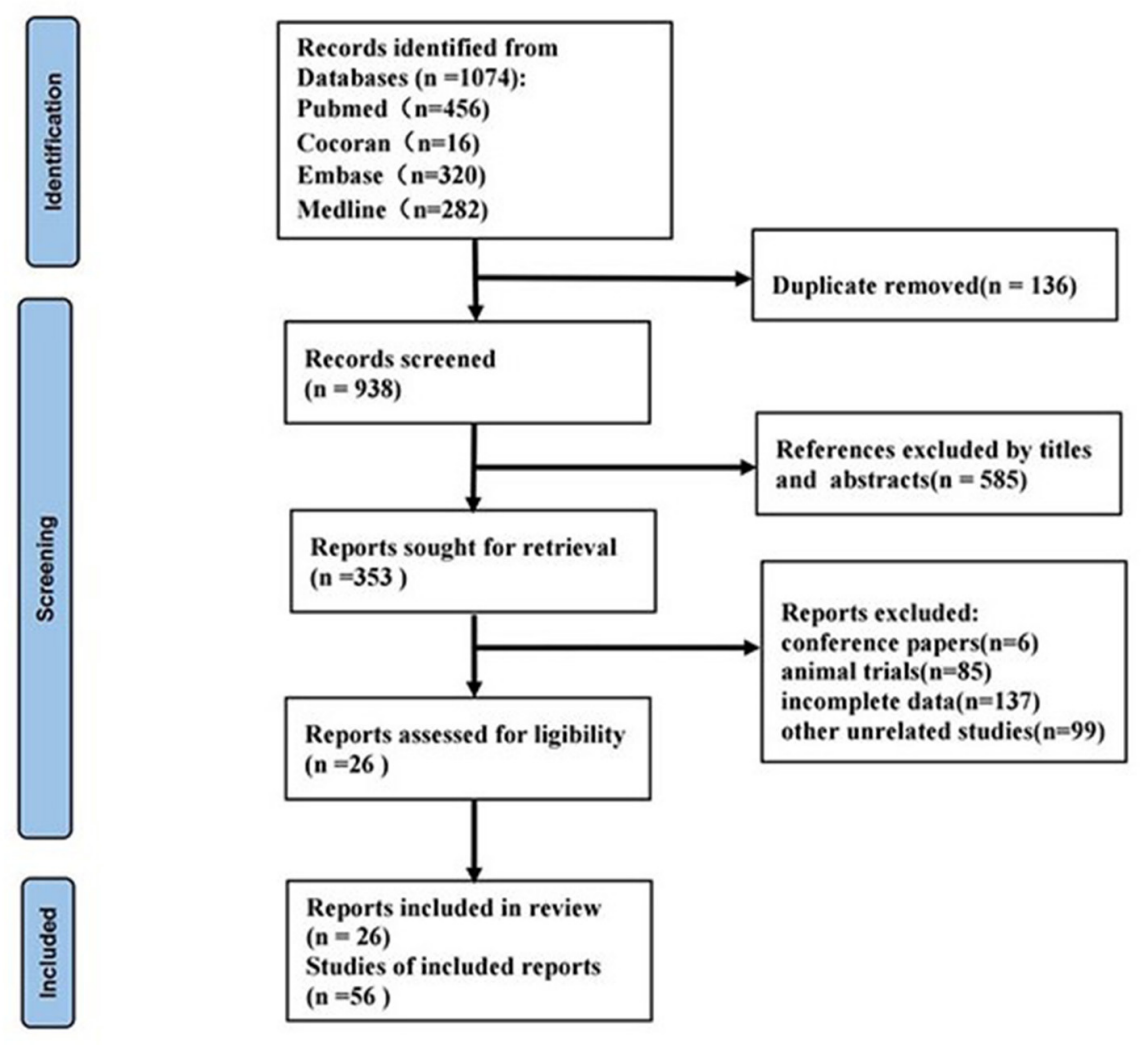
Flow diagram of the study selection.

We obtained a total of 1,074 related articles. Finally, 26 articles (11–36) were retained for full text evaluation. The flow diagram of this study was illustrated in Figure 1.

These 26 publications involved a total of 56 studies, all of which were retrospective case-control studies and included 4,098 patients with CKD and 2,450 patients without CKD. Among the 56 studies, forty-five studies reported a single miRNA and 11 studies reported miRNA panels. Detection of miRNAs was performed on serum in 7 studies, 23 studies in plasma, and 26 studies in urine. The MiRNA was detected by real-time PCR in 27 studies, 22 studies by quantitative reverse transcription polymerase chain reaction (QRT-PCR), and 7 studies by reverse transcriptase polymerase chain reaction (RT-PCR). Seventeen studies performed in Asia, 36 studies in Caucasian, and 3 studies contained both Asian and Caucasian. The patients with CKD included in the studies were involved in lupus nephritis (LN), focal segmentative glomerulonephritis (FSGS), autosomal dominant polycystic kidney disease (ADPKD), IgA nephropathy (IgAN), diabetic kidney disease (DKD), mesangial proliferative glomerulonephritis (MsPGN), and renal fibrosis (RF). Basic characters of the pieces of the literature are shown in Supplementary Materials S1, S2.

In addition, miR-150 (23, 28, 34), miR-30 (17, 22, 32), miR-192 (11, 22, 31), miR-21 (19, 23, 28), and miR-29 (14, 21, 27, 28) were studied more frequently in the included papers, so more attention could be paid to their role in CKD in the future.

In the included pieces of the literature, the panel miR-27b-3p and miR-1228-3p showed a higher diagnostic performance for DKD (15); the panels miR-106a-5p and miR-30a-5p were more accurate for MsPGN (32); the panels miR-21, miR-150, and miR-29c were superior for LN (28). Among the single miRNA, miR-21 (19), miR-192 (30), miR-636 (18), miR-34a (18, 25), and miR-342 (18) (in descending order) were more prominent in diagnosis of DKD; besides, miR-29c (28), miR-181a (13), and miR-150 (28) had strength in diagnosing LN, and miR-486-5p was preferable in IgAN.

### Quality Assessment

**Figure 2:**
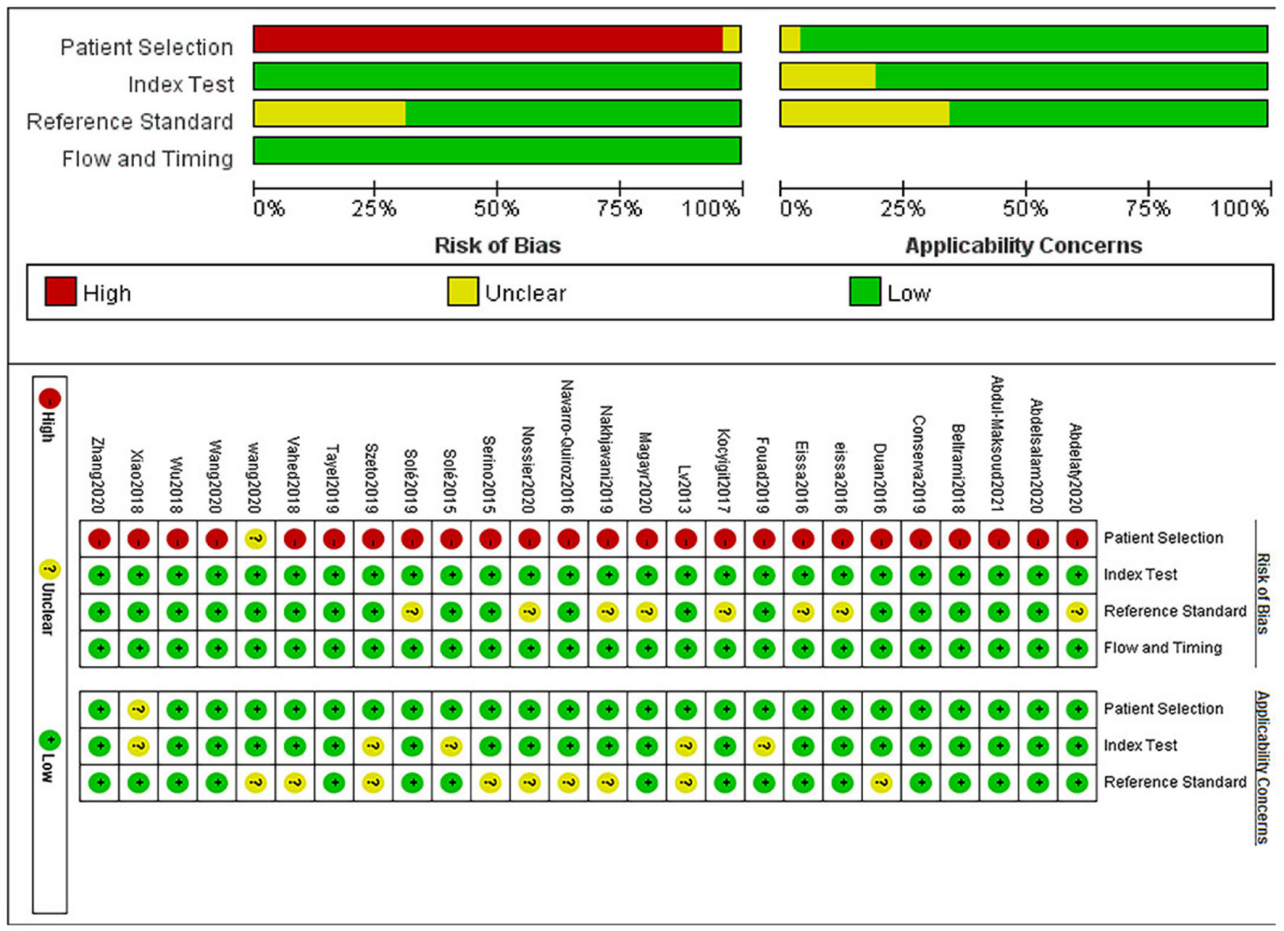
QUADAS-2 assessment of risk of bias and applicability concerns.

With the help of Revman software, the literature quality evaluation figure was drawn, as shown in Figure 2.

### Results of Meta-Analysis

#### Threshold Effect and Heterogeneity Test

The SROC curve showed no “shoulder and arm” distribution, indicating that there was no threshold effect. The PLR, NLR, sensitivity, specificity, and DOR were used as effect indicators for the statistical heterogeneity test, and the results of the study found *I*^2^ is >50, indicating a large heterogeneity among them. Therefore, the next studies would apply the random effects model.

#### Combined Sensitivity and Specificity Analysis

**Figure 3:**
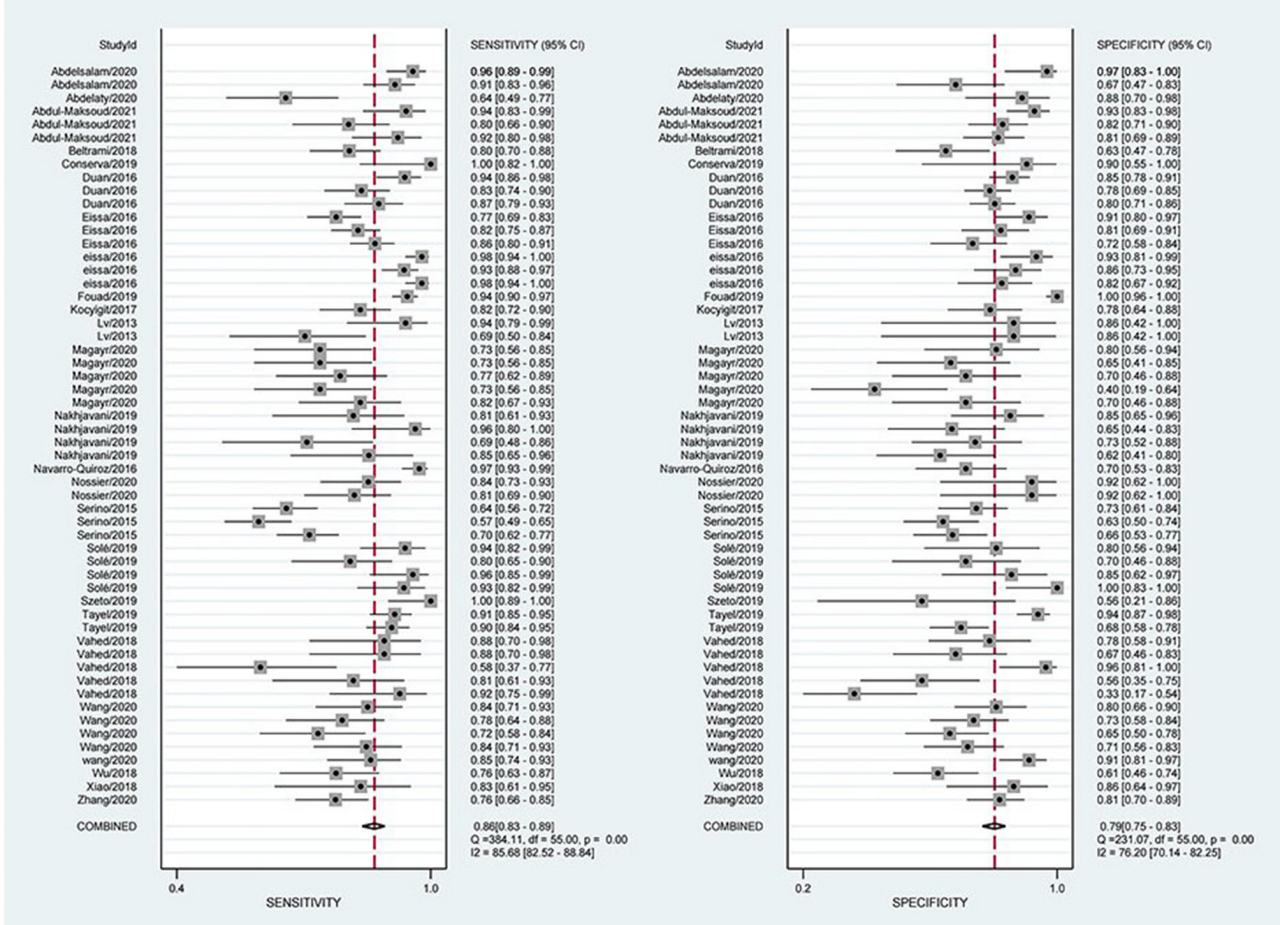
Forest plots of sensitivity and specificity on overall miRNA used in the diagnosis of CKD.

**Figure 4:**
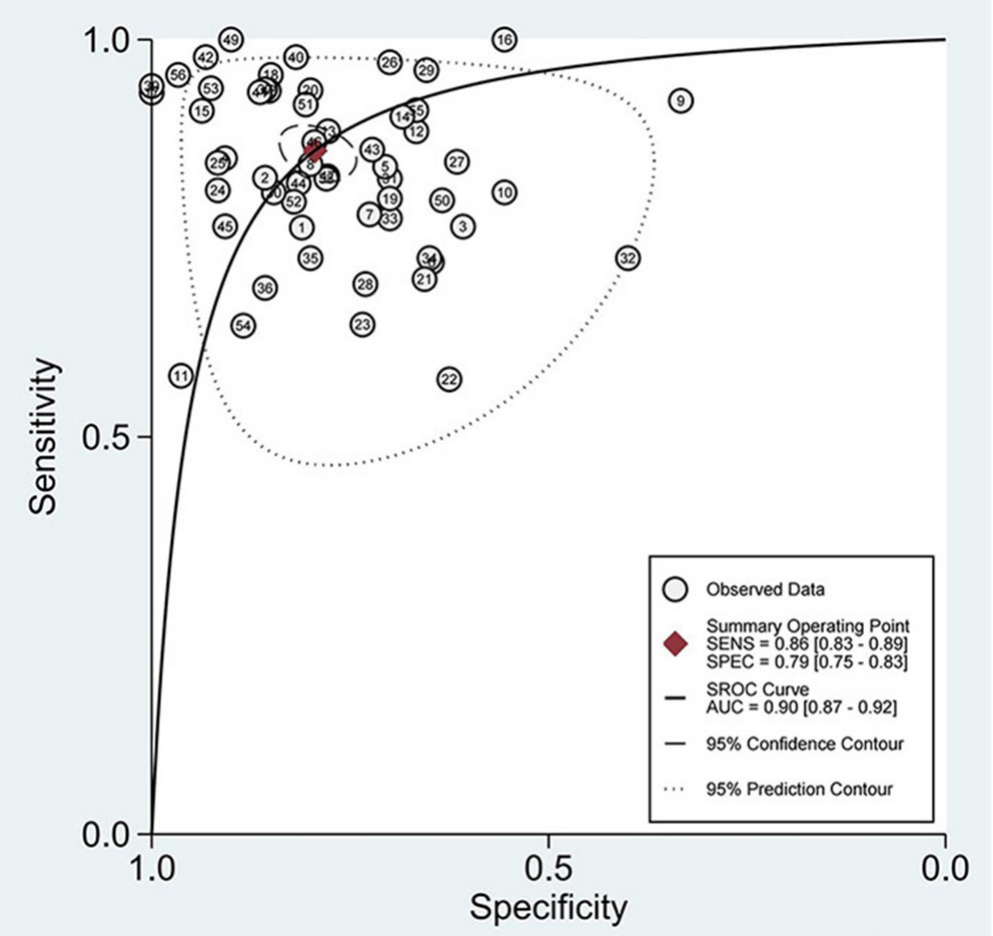
SROC curves based on all miRNAs.

The combined estimation of the diagnostic accuracy of miRNAs in CKD was as follows: sensitivity, 0.86 (95% CI: 0.83–0.89); specificity, 0.79 (95% CI: 0.75–0.83) (Figure 3); NLR, 0.18 (95% CI: 0.14–0.22); PLR, 4.19 (95% CI: 3.44–5.10); and DOR, 23.79 (95% CI: 16.39–34.55) (Supplementary Materials S5, S6). Furthermore, the AUC was 0.90 (95% CI: 0.87–0.92) (Figure 4), which indicated that miRNAs had strong accuracy and efficiency in diagnosing CKD.

#### Sensitivity Analysis, Meta-Regression Analysis, and Subgroup Analysis

**Figure 5:**
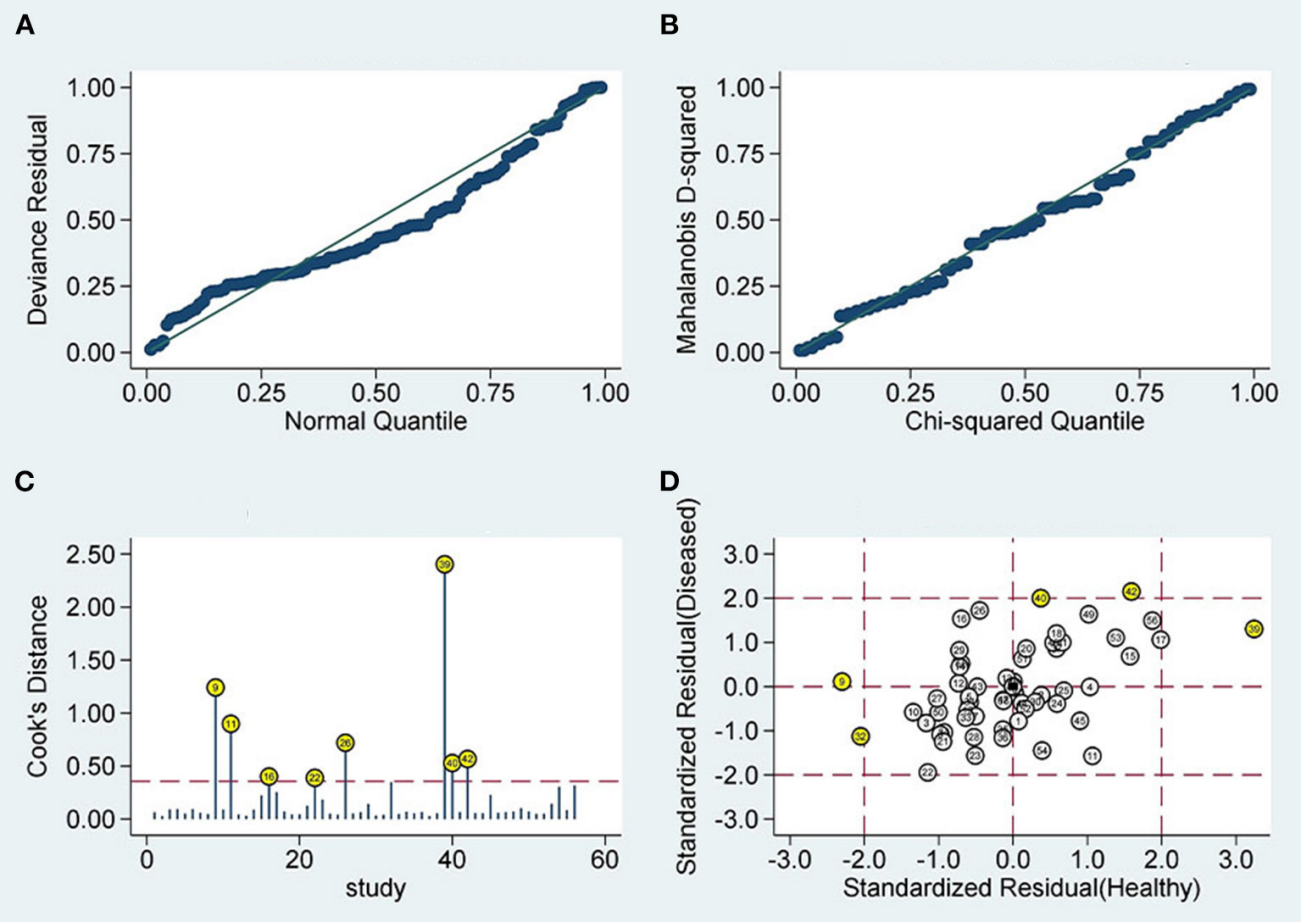
Diagram of sensitivity analysis **(A)** goodness-of-fit; **(B)** bivariate normality; **(C)** influence analysis; **(D)** outlier detection sensitivity analysis.

**Figure 6:**
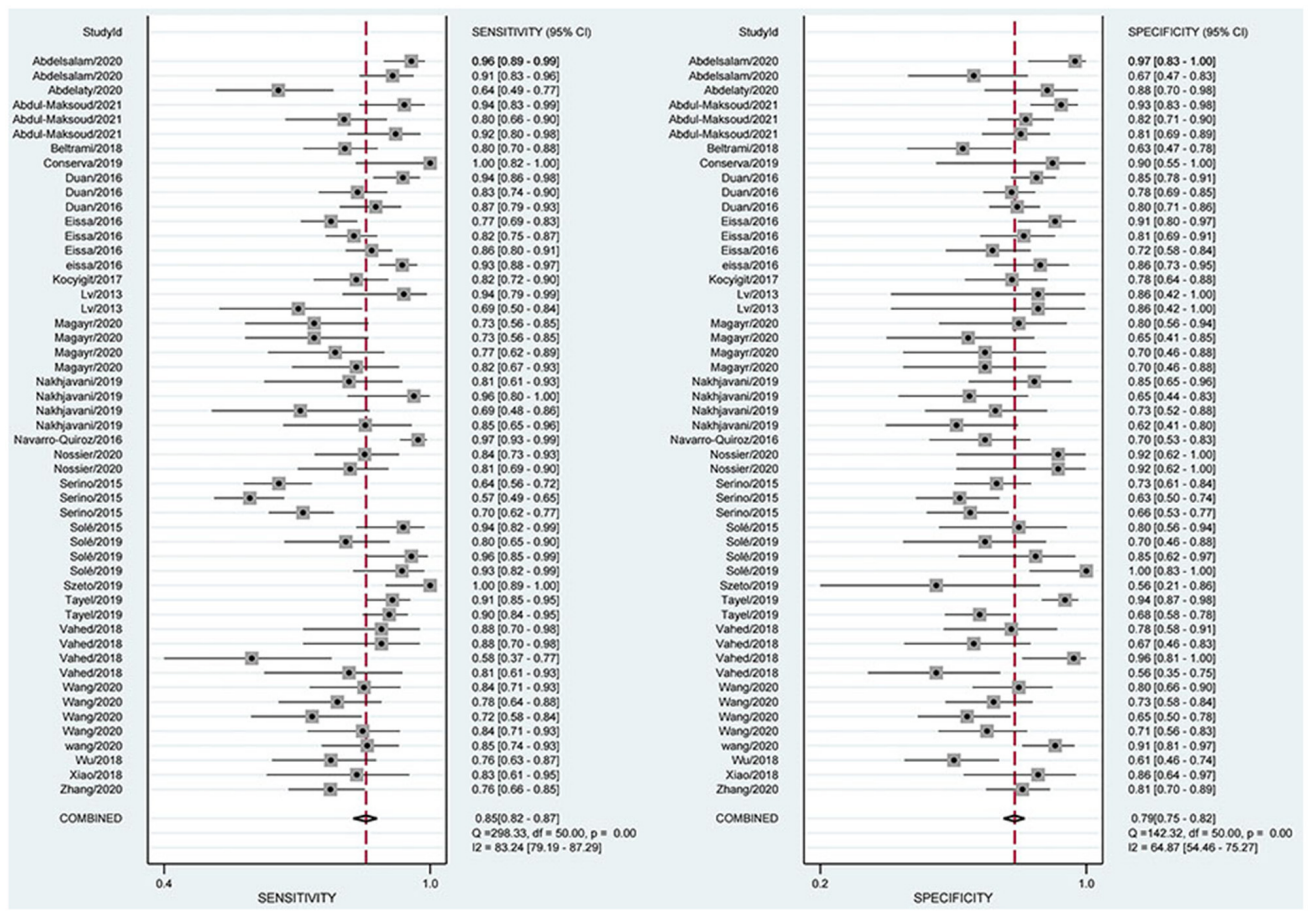
Sensitivity and specificity after deheterogeny.

Sensitivity analysis showed that the results of Goodness of Fit (Figure 5A) and bivariate normality (Figure 5B) indicated that it is reasonable to apply the random effects model analysis. The impact analysis found that four studies (24, 26, 29, 31) were the most dominant studies in weight (Figure 5C). Outlier detection suggests that four studies (18, 19, 22, 31) may be the cause of heterogeneity (Figure 5D). After excluding the five abnormal studies, the *I*^2^ value for sensitivity decreased by 2.44%, and the specificity decreased by 11.33% (Figure 6).

**Figure 7:**
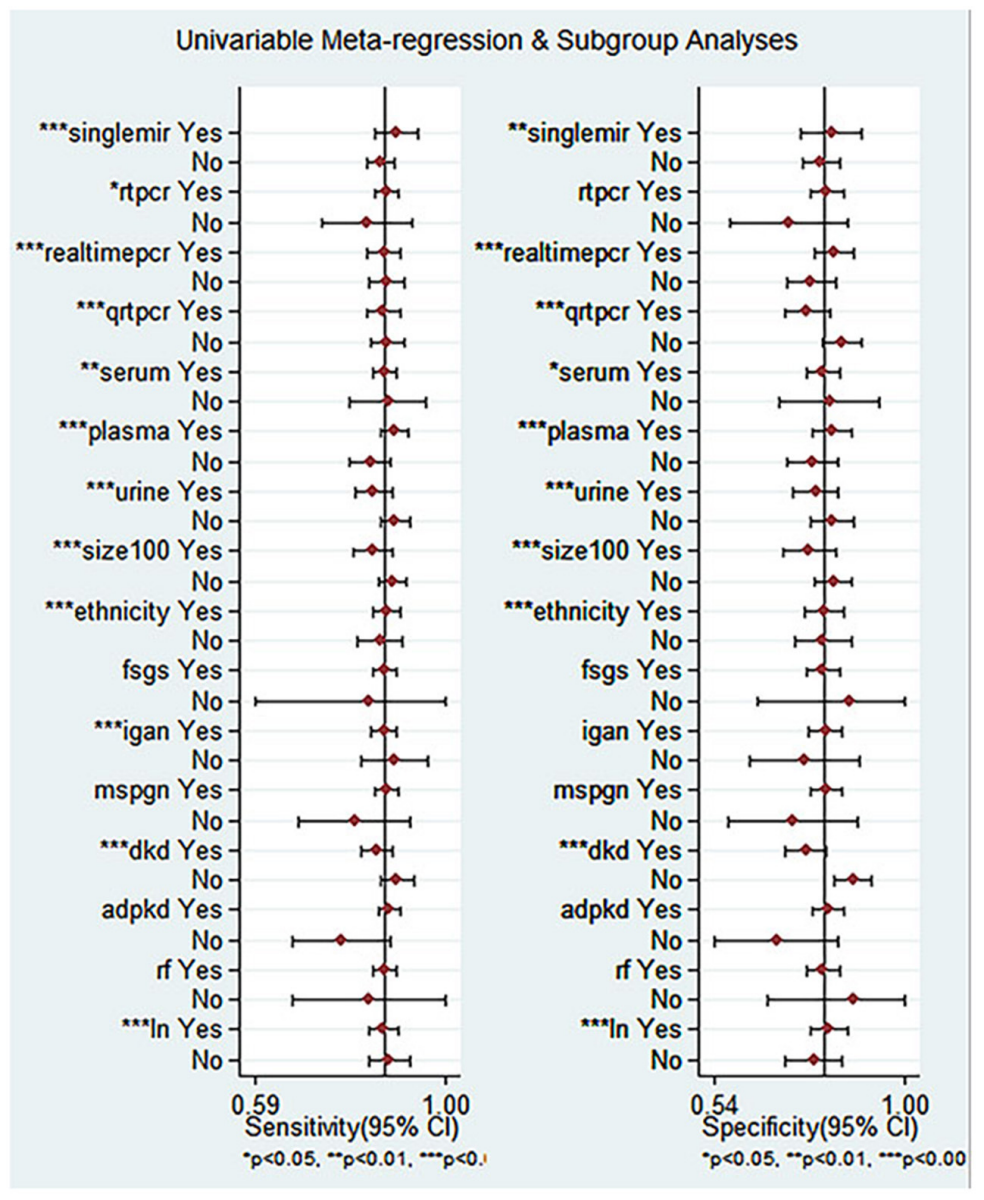
Univariable meta-reqression and subgroup analyses for sensitivity and specificity of miRNA for diagnosis of CKD.

To further uncover the heterogeneity among these studies, we undertook a regression analysis and a subgroup analysis based on ethnicity, miRNA profiling, method of detection, sample size, tissue, and disease spectrum. Some of the results are shown in Supplementary Material S3. Since three studies (26) included both Asians and Caucasians, regression analyses on ethnicity were performed after excluding them (Figure 7).

**Figure 8:**
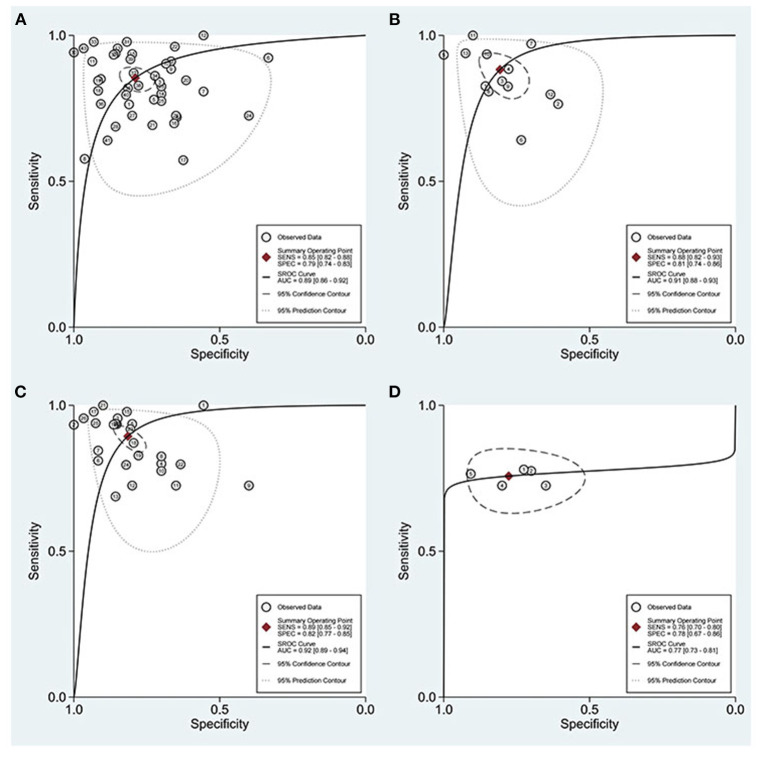
ROC curves based on miRNAs. **(A)** Single miRNA; **(B)** miRNAs panel; **(C)** miRNAs detected in urine; **(D)** miRNA-30.

Compared with single miRNA, the diagnostic accuracy of the miRNAs panel is relatively outstanding, with sensitivity of 0.85 vs. 0.88, specificity of 0.79 vs. 0.81, PLR of 4.06 vs. 4.58, NLR of 0.18 vs. 0.15, DOR of 22 vs. 32, and AUC of 0.89 vs. 0.91, respectively (Figures 8A,B). The sensitivity of Asians was 0.85, specificity was 0.76, and AUC was 0.88, compared with which miRNAs have higher overall diagnostic accuracy in Caucasians, with sensitivity of 0.87, specificity of 0.79, and AUC of 0.90, respectively.

With regard to blood, on the one hand, the plasma was 0.84 for sensitivity, 0.78 for specificity, 3.8 for PLR, 0.21 for NLR, 19 for DOR, and 0.88 for AUC; on the other hand, miRNAs in serum were 0.79, 0.76, 3.3, 0.27, 12, and 0.83, respectively. For urine, the sensitivity, specificity, PLR, NLR, DOR, and AUC were 0.89, 0.82, 4.8, 0.13, 37, and 0.92, respectively (Figure 8C), suggesting that miRNAs in urine have superior diagnostic performance rather than in blood.

The results of the disease analysis showed favorable accuracy for all diseases, while DKD had the highest diagnostic accuracy. There were 17 studies on DKD, and their sensitivity, specificity, PLR, NLR, DOR, and AUC of DKD were 0.90, 0.88, 7.5, 0.12, 64, and 0.95, respectively. For the reason of the small sample size, MsPGN, FSGS, and RF were not analyzed separately in this meta-analysis for the time being. Due to the fact that the miRNA30 family was reported in five studies, a separate analysis of miRNA-30 was performed (Figure 8D). The sensitivity and specificity of miR-30 were 0.76 (95% CI: 0.70–0.80) and 0.78 (95% CI: 0.67–0.86), respectively, while the PLR, NLR, DOR, and AUC were 3.4 (95% CI: 2.2–5.2), 0.31 (95% CI: 0.24–0.40), 11 (95% CI: 6–20), and 0.77 (95% CI: 0.73–0.81), respectively.

#### Analysis of Publication Bias

**Figure 9:**
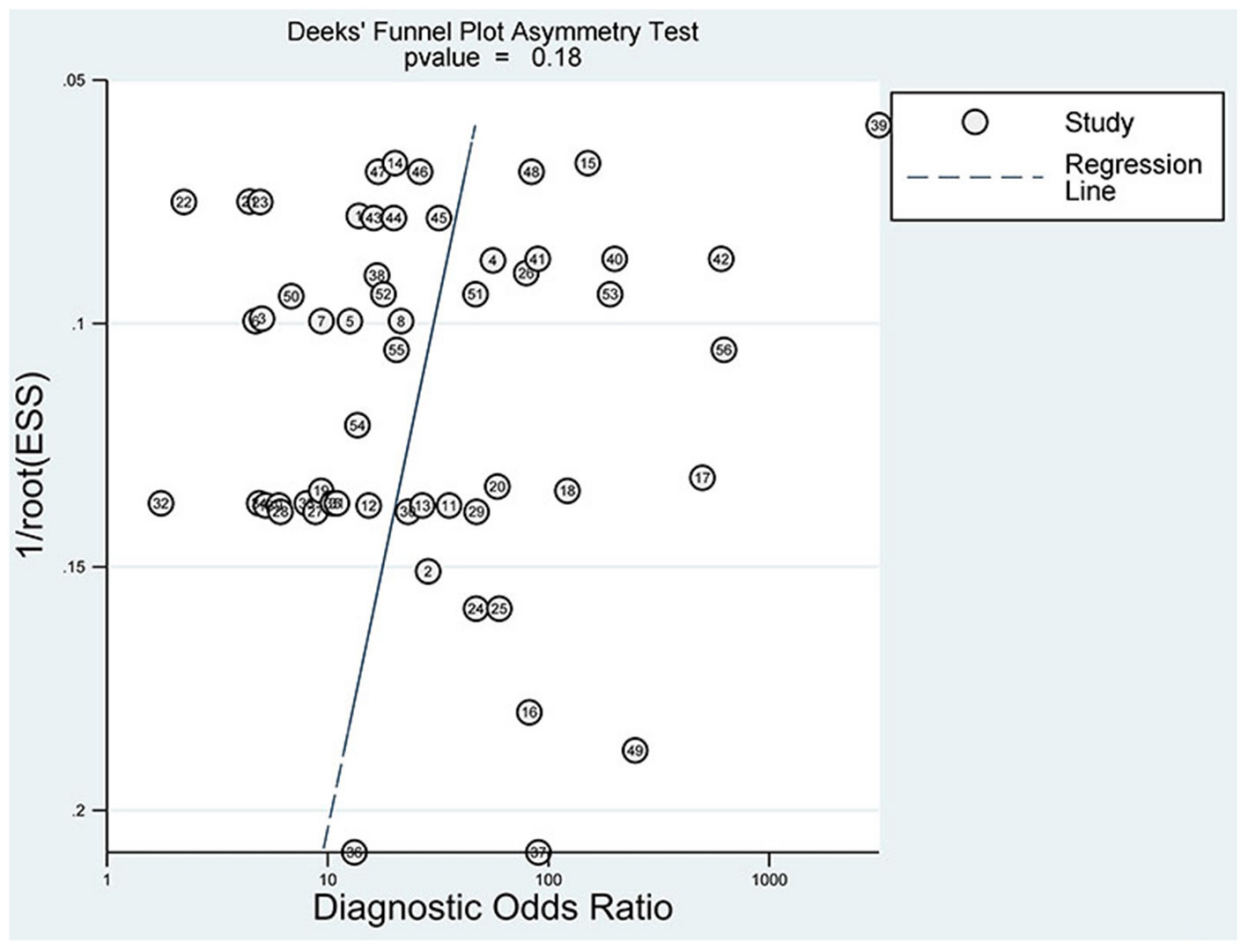
Funnel plot of publication bias.

The slope coefficient *P*-value of the funnel plot (Figure 9) is 0.18 > 0.05, demonstrating that no significant publication bias exists.

## Discussion

As CKD is a silent disease, the search for more convenient and accurate biomarkers and assays has been a high concern for clinicians, so more and more trials on miRNAs and CKD have been conducted or are in progress. In this meta-analysis of 56 studies involving 4,098 patients with CKD and 2,450 patients without CKD, miRNA remained highly sensitive (0.86) and highly specific (0.79) in the diagnosis of CKD. We plotted the SROC curve, and the AUC was 0.90.

Helping clinical diagnosis is one of the essential values of biomarkers. Likelihood ratios are instructive to clinicians because they provide a reference about the possibility of a positive or negative patient progressing to CKD. The PLR of 4.19 demonstrated a 4-fold improvement in the ability to diagnose CKD correctly, while the NLR of 0.18 demonstrated an 82% decrease in the probability of successful diagnosis of CKD when miRNAs expression was reversed. At the same time, the DOR of miRNAs (23.79) also showed a high diagnostic capability, which also confirmed that the detection of miRNAs may be a necessary means for the diagnosis and monitoring of CKD in the future. We also mapped a graph (Supplementary Material S7) about the combined PLR and NLR to determine their clinical applicability. When the publications are in the first quadrant, namely LRP >10 and LRN <0.1, the results revealed high applicability. We discovered that miRNAs in four studies (17, 30, 32, 36) had superior diagnostic efficacy. Therefore, a single miRNA (miR-133, miR-30a, miR-126, etc.) and the panel of miRNAs, such as panels miR-106a-5p and miR-30a-5p, may be more promising and deserve future research.

Subgroup analysis showed that CKD had an excellent accuracy in plasma, serum, and urine samples, and yet the highest in urine samples, with the sensitivity, specificity, and AUC of 0.89, 0.82 and 0.92, respectively. Many studies have found that miRNA is stable and promising in both blood and urine tests, but the biomarkers in urine are better (37), which is consistent with our results. The MiRNAs not only exist widely in human tissues and cells but can also be stably expressed in plasma, serum, and other body fluids by binding with Argonaute protein to prevent the degradation of RNA enzymes. This stability makes the miRNAs possible to become the biomarkers for disease diagnosis (38). In addition, a massive study that analyzed the relationship between miRNA expression in dozens of healthy human tissues and body fluids, such as plasma, serum, and urine, etc., showed that miRNAs in urine correlated most closely with miRNAs in the kidney (39). Given that the use of urine specimens can provide a non-invasive, safe, and convenient way to obtain important diagnostic information, urine is apparently attractive to the clinics. Bidin et al. compared blood and urine samples from 63 publications to assess the superiority of both as an early diagnosis or monitoring of CKD, and the results suggest that urine is more advantageous as a biomarker compared to blood (7).

Furthermore, many studies have found that miRNAs detected in urine have diagnostic and differential diagnostic implications for many kidney diseases. Ben-Dov et al. screened miRNAs in renal epithelial cells and urine samples in ADPKD and non-ADPKD, and identified miR-1(4) and mir-133b (2) in ADPKD, suggesting that they could be used as biomarkers to monitor the progression of ADPKD (40). Conserva et al. concluded that the expression of miR-27b-3p and miR-1228-3p was downregulated in both biopsies and urine, and that expression in urine was also capable of identifying patients with DN from other glomerulonephritis (15).

Of course, in addition to being valuable for the diagnosis of CKD, miRNAs provide new ways to elucidate the underlying mechanisms of CKD and have an increasingly prominent role in therapy, which, therefore, may become a new promising therapeutic target (41, 42). The activity of specific miRNAs in the kidney can be achieved by delivering mimics *in vivo* to restore the miRNA levels or by inhibitors to block miRNA function (43). It was found that miRNA-21 expression was increased in unilateral ureteral obstruction (UUO) or ischemic reperfusion injury (IRI) models and that blocking miRNA-21 *in vivo* could attenuate the induced renal fibrosis (44, 45). Bao et al. (46) found that the inhibition of miR-21 activation by blocking the PTEN/Akt pathway in IgAN prevented the fibrogenic activation in podocytes and renal tubular cells. Liao et al. (47) showed that miR-140-5p can protect HK2 cells from TGF-β-induced renal fibrosis by directly targeting TGFBR1. In a word, we cannot ignore the fact that miRNA as a biomarker of CKD should be put into more research.

### Strength and Limitations

These are main merits of this meta-analysis: (1) It is the first meta-analysis to summarize at length the value of miRNAs in the diagnosis of CKD; (2) This meta found the superiority of miRNAs in urine specimens for the diagnosis of CKD rather than serum and plasma; and (3) Macroscopically, it incorporates more comprehensive miRNA types; in terms of details, it also made a careful analysis of each included literature and listed specific miRNAs' subtypes that are worthy of more research in the future.

However, this review may carry the following shortcomings: (1) The included pieces of literature only include papers published in English, which may exclude the pieces of literature in other languages, leading to inevitable errors; (2) Due to the lack of data, the diagnostic value of miRNA for different grades of CKD was not evaluated. The subgroup analysis included diseases, such as DKD, IgAN, and LN, and other CKD diseases were not studied in detail, so further studies should include these issues; (3) There is a lack of a uniform critical level, so there is an obvious gap of sample size between the experimental and control groups in some studies; and (4) Most pieces of literature do not explain the threshold set before the miRNA detection, and the detection time points are different, which may affect the implementation of detection, and then affect the strength of the demonstration of the results. More clinical case-control data are required in the future to verify the reliability of the analysis results.

## Conclusions

Our study indicates that miRNA is of great diagnostic value in CKD and may become an effective non-invasive biological marker for CKD. Additionally, miRNA panels had higher diagnostic potency than single miRNA. What is more, miRNAs in both blood and urine have significant accuracy in the diagnosis of CKD; nevertheless, urine is superior. However, high-quality and large-scale studies are still needed to reveal the exact relationship between miRNAs and CKD.

## Author Contributions

JL and LM conducted independently the article search and quality assessment, wrote the manuscript, and conceived the study. Any disagreements between the two researchers were settled through discussions with the other two researchers (HYu and YY). HYu and YY extracted the data. ZX and WL performed the statistical analysis. HYa assessed the quality of results of this study. LW and XW revised the paper for English. All authors critically reviewed the report. All authors contributed to the article and approved the submitted version.

## Funding

This study was supported by the National Key Research and Development Program of China (Reference No. 2019YFC1709401), the National Natural Science Foundation of China (Reference No. 82004316), and the National Natural Science Foundation of China (Reference No. 81973799).

## Conflict of Interest

The authors declare that the research was conducted in the absence of any commercial or financial relationships that could be construed as a potential conflict of interest.

## Publisher's Note

All claims expressed in this article are solely those of the authors and do not necessarily represent those of their affiliated organizations, or those of the publisher, the editors and the reviewers. Any product that may be evaluated in this article, or claim that may be made by its manufacturer, is not guaranteed or endorsed by the publisher.
