# Supplementary material for: MicroRNAs as Potential Biomarkers for the Diagnosis of Chronic Kidney Disease: A Systematic Review and Meta-Analysis
Source: Front Med (Lausanne). 2022 Feb 7;8:782561. doi: 10.3389/fmed.2021.782561 (PMC8860181; doi:10.3389/fmed.2021.782561)
Supplement: Supplementary file 1 [file Data_Sheet_1.docx]

**S1 Characteristics of studies included in the present meta-analysis**

| author（year） | ethnicity | disease | detection | tissues | sample size |
| --- | --- | --- | --- | --- | --- |
| Abdelaty2020 | Caucasian | DKD | qRT-PCR | plasma | 75 |
| Abdelsalam2020 | Caucasian | DKD | qRT-PCR | plasma | 120 |
| Abdelsalam2020 | Caucasian | DKD | qRT-PCR | urine | 120 |
| Abdul-Maksoud2021 | Caucasian | LN | Real-time PCR | urine | 116 |
| Abdul-Maksoud2021 | Caucasian | LN | Real-time PCR | urine | 116 |
| Abdul-Maksoud2021 | Caucasian | LN | Real-time PCR | urine | 116 |
| Beltrami2018 | Caucasian | DKD | qRT-PCR | urine | 130 |
| Conserva2019 | Caucasian | DKD | Real-time PCR | urine | 29 |
| Duan2016 | Asian | IgAN | Real-time PCR | urine | 215 |
| Duan2016 | Asian | IgAN | Real-time PCR | urine | 215 |
| Duan2016 | Asian | IgAN | Real-time PCR | urine | 215 |
| Eissa2016 | Caucasian | DKD | qRT-PCR | plasma | 220 |
| Eissa2016 | Caucasian | DKD | qRT-PCR | plasma | 220 |
| Eissa2016 | Caucasian | DKD | qRT-PCR | plasma | 220 |
| eissa2016 | Caucasian | DKD | Real-time PCR | urine | 180 |
| eissa2016 | Caucasian | DKD | Real-time PCR | urine | 180 |
| eissa2016 | Caucasian | DKD | Real-time PCR | urine | 180 |
| Fouad2019 | Caucasian | DKD | qRT-PCR | plasma | 340 |
| Kocyigit2017 | Caucasian | ADPKD | qRT-PCR | Serum | 130 |
| Lv2013 | Asian | CKD | qRT-PCR | urine | 39 |
| Lv2013 | Asian | CKD | qRT-PCR | urine | 39 |
| Magayr2020 | Caucasian | ADPKD | Real-time PCR | urine | 60 |
| Magayr2020 | Caucasian | ADPKD | Real-time PCR | urine | 60 |
| Magayr2020 | Caucasian | ADPKD | Real-time PCR | urine | 60 |
| Magayr2020 | Caucasian | ADPKD | Real-time PCR | urine | 60 |
| Magayr2020 | Caucasian | ADPKD | Real-time PCR | urine | 60 |
| Nakhjavani2019 | Caucasian | LN | Real-time PCR | Plasma | 52 |
| Nakhjavani2019 | Caucasian | LN | Real-time PCR | Plasma | 52 |
| Nakhjavani2019 | Caucasian | LN | Real-time PCR | Plasma | 52 |
| Nakhjavani2019 | Caucasian | LN | Real-time PCR | Plasma | 52 |
| Navarro-Quiroz2016 | Caucasian | LN | qRT-PCR | serum | 180 |
| Nossier2020 | Caucasian | DKD | qRT-PCR | urine | 70 |
| Nossier2020 | Caucasian | DKD | qRT-PCR | urine | 70 |
| Serino2015 | Caucasian/Asian | IgAN | Real-time PCR | serum | 209 |
| Serino2015 | Caucasian/Asian | IgAN | Real-time PCR | serum | 209 |
| Serino2015 | Caucasian/Asian | IgAN | Real-time PCR | serum | 209 |
| Solé2015 | Caucasian | LN | qRT-PCR | urine | 67 |
| Solé2019 | Asian | LN | qRT-PCR | urine | 65 |
| Solé2019 | Asian | LN | qRT-PCR | urine | 65 |
| Solé2019 | Asian | LN | qRT-PCR | urine | 65 |
| Szeto2019 | Asian | IgAN | qRT-PCR | urine | 42 |
| Tayel2019 | Caucasian | DKD | RT-PCR | plasma | 229 |
| Tayel2019 | Caucasian | DKD | qRT-PCR | plasma | 229 |
| Vahed2018 | Caucasian | LN | Real-time PCR | plasma | 53 |
| Vahed2018 | Caucasian | LN | Real-time PCR | plasma | 53 |
| Vahed2018 | Caucasian | LN | Real-time PCR | plasma | 53 |
| Vahed2018 | Caucasian | LN | Real-time PCR | plasma | 53 |
| Vahed2018 | Caucasian | LN | Real-time PCR | plasma | 53 |
| Wang2020 | Asian | MsPGN | RT-PCR | plasma | 100 |
| Wang2020 | Asian | MsPGN | RT-PCR | plasma | 100 |
| Wang2020 | Asian | MsPGN | RT-PCR | plasma | 100 |
| Wang2020 | Asian | MsPGN | RT-PCR | plasma | 100 |
| wang2020 | Asian | DKD | qRT-PCR | serum | 132 |
| Wu2018 | Asian | IgAN | qRT-PCR | plasma | 102 |
| Xiao2018 | Asian | FSGS | qRT-PCR | plasma | 44 |
| Zhang2020 | Asian | LN | qRT-PCR | serum | 167 |

**S2 Basic information about the literature on diagnostic efficacy**

| author（year） | miRNA | TP | FP | FN | TN | sensitivity | specificity |
| --- | --- | --- | --- | --- | --- | --- | --- |
| Abdelaty2020 | miR-192 | 32 | 3 | 18 | 23 | 0.63 | 0.90 |
| Abdelsalam2020 | miR-451 | 82 | 10 | 8 | 20 | 0.91 | 0.67 |
| Abdelsalam2020 | miR-451 | 86 | 1 | 4 | 29 | 0.96 | 0.96 |
| Abdul-Maksoud2021 | miR-181a | 45 | 13 | 4 | 54 | 0.92 | 0.81 |
| Abdul-Maksoud2021 | miR-223 | 39 | 12 | 10 | 55 | 0.79 | 0.83 |
| Abdul-Maksoud2021 | panel：miR-181a,miR-223 | 46 | 5 | 3 | 62 | 0.94 | 0.92 |
| Beltrami2018 | panel：miR-29b,miR-126-3p,miR-155-5p | 71 | 15 | 18 | 26 | 0.80 | 0.63 |
| Conserva2019 | panel：miR-27b-3p,miR-1228-3p | 19 | 1 | 0 | 9 | 1.00 | 0.90 |
| Duan2016 | miR-25-3p | 81 | 25 | 12 | 97 | 0.87 | 0.79 |
| Duan2016 | miR-144-3p | 77 | 27 | 16 | 95 | 0.82 | 0.78 |
| Duan2016 | miR-486-5p | 87 | 18 | 6 | 104 | 0.94 | 0.85 |
| Eissa2016 | miR-133b | 143 | 15 | 23 | 39 | 0.86 | 0.72 |
| Eissa2016 | miR-342 | 136 | 10 | 30 | 44 | 0.82 | 0.81 |
| Eissa2016 | miR-30a | 127 | 5 | 39 | 49 | 0.76 | 0.91 |
| eissa2016 | miR-15b | 133 | 8 | 3 | 36 | 0.98 | 0.82 |
| eissa2016 | miR-34a | 127 | 6 | 9 | 38 | 0.93 | 0.86 |
| eissa2016 | miR-636 | 133 | 3 | 3 | 41 | 0.98 | 0.93 |
| Fouad2019 | miR-21 | 226 | 0 | 14 | 100 | 0.94 | 1.00 |
| Kocyigit2017 | miR-3907 | 66 | 11 | 14 | 39 | 0.83 | 0.78 |
| Lv2013 | miR-29c | 22 | 1 | 10 | 6 | 0.69 | 0.81 |
| Lv2013 | miR-29a | 30 | 1 | 2 | 6 | 0.94 | 0.81 |
| Magayr2020 | miR-192-5p | 33 | 6 | 7 | 14 | 0.82 | 0.70 |
| Magayr2020 | miR-194-5p | 29 | 12 | 11 | 8 | 0.72 | 0.41 |
| Magayr2020 | miR-30a-5p | 31 | 6 | 9 | 14 | 0.77 | 0.70 |
| Magayr2020 | miR-30d-5p | 29 | 7 | 11 | 13 | 0.73 | 0.65 |
| Magayr2020 | miR-30e-5p | 29 | 4 | 11 | 16 | 0.73 | 0.82 |
| Nakhjavani2019 | miR-21 | 22 | 10 | 4 | 16 | 0.86 | 0.63 |
| Nakhjavani2019 | miR-150 | 18 | 7 | 8 | 19 | 0.69 | 0.72 |
| Nakhjavani2019 | miR-423 | 25 | 9 | 1 | 17 | 0.96 | 0.66 |
| Nakhjavani2019 | panel：miR-21,miR-150,miR-423 | 21 | 4 | 5 | 22 | 0.79 | 0.83 |
| Navarro-Quiroz2016 | panel：miR-221-5p,miR-380-3p,miR-556-5p,miR-758-3p,miR-3074-3p | 136 | 12 | 4 | 28 | 0.97 | 0.70 |
| Nossier2020 | miR-210 | 47 | 1 | 11 | 11 | 0.81 | 0.88 |
| Nossier2020 | miR-34a | 49 | 1 | 9 | 11 | 0.84 | 0.94 |
| Serino2015 | let7b | 102 | 22 | 44 | 42 | 0.70 | 0.66 |
| Serino2015 | miR-148b | 83 | 24 | 62 | 40 | 0.57 | 0.63 |
| Serino2015 | panel：miR-148b,let7b | 93 | 17 | 52 | 47 | 0.64 | 0.74 |
| Solé2015 | miR-29c | 44 | 4 | 3 | 16 | 0.94 | 0.82 |
| Solé2019 | panel：miR-21,miR-150,miR-29c | 42 | 0 | 3 | 20 | 0.94 | 0.99 |
| Solé2019 | miR-150 | 43 | 3 | 2 | 17 | 0.96 | 0.83 |
| Solé2019 | miR-21 | 36 | 6 | 9 | 14 | 0.81 | 0.72 |
| Szeto2019 | miR-204 | 33 | 4 | 0 | 5 | 1.00 | 0.56 |
| Tayel2019 | miR-126 | 121 | 30 | 12 | 65 | 0.91 | 0.68 |
| Tayel2019 | miR-192 | 122 | 6 | 12 | 89 | 0.91 | 0.94 |
| Vahed2018 | miR-125a | 24 | 18 | 2 | 9 | 0.92 | 0.33 |
| Vahed2018 | miR-142-3p | 21 | 12 | 5 | 15 | 0.81 | 0.56 |
| Vahed2018 | miR-146 | 15 | 1 | 11 | 26 | 0.56 | 0.96 |
| Vahed2018 | miR-156 | 23 | 9 | 3 | 18 | 0.88 | 0.67 |
| Vahed2018 | panel：miR-125a,miR-142-3p,miR-146,miR-156 | 23 | 6 | 3 | 21 | 0.89 | 0.79 |
| Wang2020 | miR-106a-5p | 42 | 15 | 8 | 36 | 0.84 | 0.71 |
| Wang2020 | miR-17-5p | 36 | 18 | 14 | 33 | 0.72 | 0.65 |
| Wang2020 | miR-30a-5p | 39 | 14 | 11 | 37 | 0.78 | 0.73 |
| Wang2020 | panel：miR-106a-5p,miR-30a-5p | 43 | 10 | 8 | 40 | 0.85 | 0.80 |
| wang2020 | miR-16-5p | 57 | 6 | 10 | 59 | 0.85 | 0.91 |
| Wu2018 | panel：miR-148a-3p, miR-150-5p, miR-20a-5p,miR-425-3p | 39 | 20 | 12 | 31 | 0.77 | 0.61 |
| Xiao2018 | panel：miR-17,miR-451,miR-106a,miR-19b | 19 | 3 | 4 | 18 | 0.83 | 0.85 |
| Zhang2020 | miR-203 | 71 | 14 | 22 | 60 | 0.76 | 0.81 |

TP:true positive,FP:false positive,FN:false negative,TN:true negative

| Subgroup | | sensitivity | specificity | PLR | NLR | DOR | AUC |
| --- | --- | --- | --- | --- | --- | --- | --- |
| miRNA | single | 0.86 | 0.79 | 4.1 | 0.18 | 22 | 0.89 |
|  | panel | 0.88 | 0.81 | 4.6 | 0.15 | 31 | 0.91 |
| ethnicity | Caucasians | 0.88 | 0.81 | 4.5 | 0.15 | 29 | 0.91 |
|  | Asians | 0.85 | 0.79 | 4.1 | 0.19 | 22 | 0.89 |
| tissue | urine | 0.89 | 0.82 | 4.8 | 0.13 | 37 | 0.92 |
|  | plasma | 0.87 | 0.78 | 3.8 | 0.21 | 19 | 0.88 |
|  | serum | 0.79 | 0.76 | 3.3 | 0.27 | 12 | 0.83 |
| disease | DKD | 0.9 | 0.88 | 7.5 | 0.12 | 64 | 0.95 |
|  | LN | 0.88 | 0.78 | 4.0 | 0.15 | 26 | 0.90 |
|  | IgAN | 0.81 | 0.74 | 3.2 | 0.25 | 13 | 0.82 |
|  | ADPKD | 0.77 | 0.68 | 2.4 | 0.33 | 7 | 0.80 |
| miRNA30 |  | 0.76 | 0.78 | 3.4 | 3.10 | 11 | 0.77 |

**S3 Results of partial subgroup analysis（PLR:positive likelihood ratio,NLR:negative likelihood ratio , DOR:diagnostic odds ratio）**

**S4 Search strategy**

**Pubmed:**("kidney diseases"[MeSH Terms] OR "kidney failure"[Title/Abstract] OR "kidney insufficiency"[Title/Abstract] OR "kidney function"[Title/Abstract] OR "kidney dysfunction"[Title/Abstract] OR "renal disease"[Title/Abstract] OR "renal failure"[Title/Abstract] OR "renal insufficiency"[Title/Abstract] OR "renal function"[Title/Abstract] OR "renal dysfunction"[Title/Abstract] OR "nephritis"[Title/Abstract] OR "chronic renal failure"[Title/Abstract] OR "CKD"[Title/Abstract]) AND ("micrornas"[MeSH Terms] OR "MicroRNA"[Title/Abstract] OR "miRNAs"[Title/Abstract] OR "micro rna"[Title/Abstract] OR "rna micro"[Title/Abstract] OR "miRNA"[Title/Abstract] OR "primary microrna"[Title/Abstract] OR "microrna primary"[Title/Abstract] OR "primary mirna"[Title/Abstract] OR "mirna primary"[Title/Abstract] OR "pri-miRNA"[Title/Abstract] OR "pri-miRNA"[Title/Abstract] OR (("RNA"[MeSH Terms] OR "RNA"[All Fields]) AND "small temporal"[Title/Abstract]) OR (("Temporal"[All Fields] OR "temporally"[All Fields] OR "temporals"[All Fields]) AND "rna small"[Title/Abstract]) OR "stRNA"[Title/Abstract] OR "small temporal rna"[Title/Abstract] OR "pre-miRNA"[Title/Abstract] OR "pre-miRNA"[Title/Abstract]) AND ("diagnosis"[Title/Abstract] OR "predict"[Title/Abstract] OR "sensitivity"[Title/Abstract] OR "specificity"[Title/Abstract] OR "ROC"[Title/Abstract] OR "AUC"[Title/Abstract])


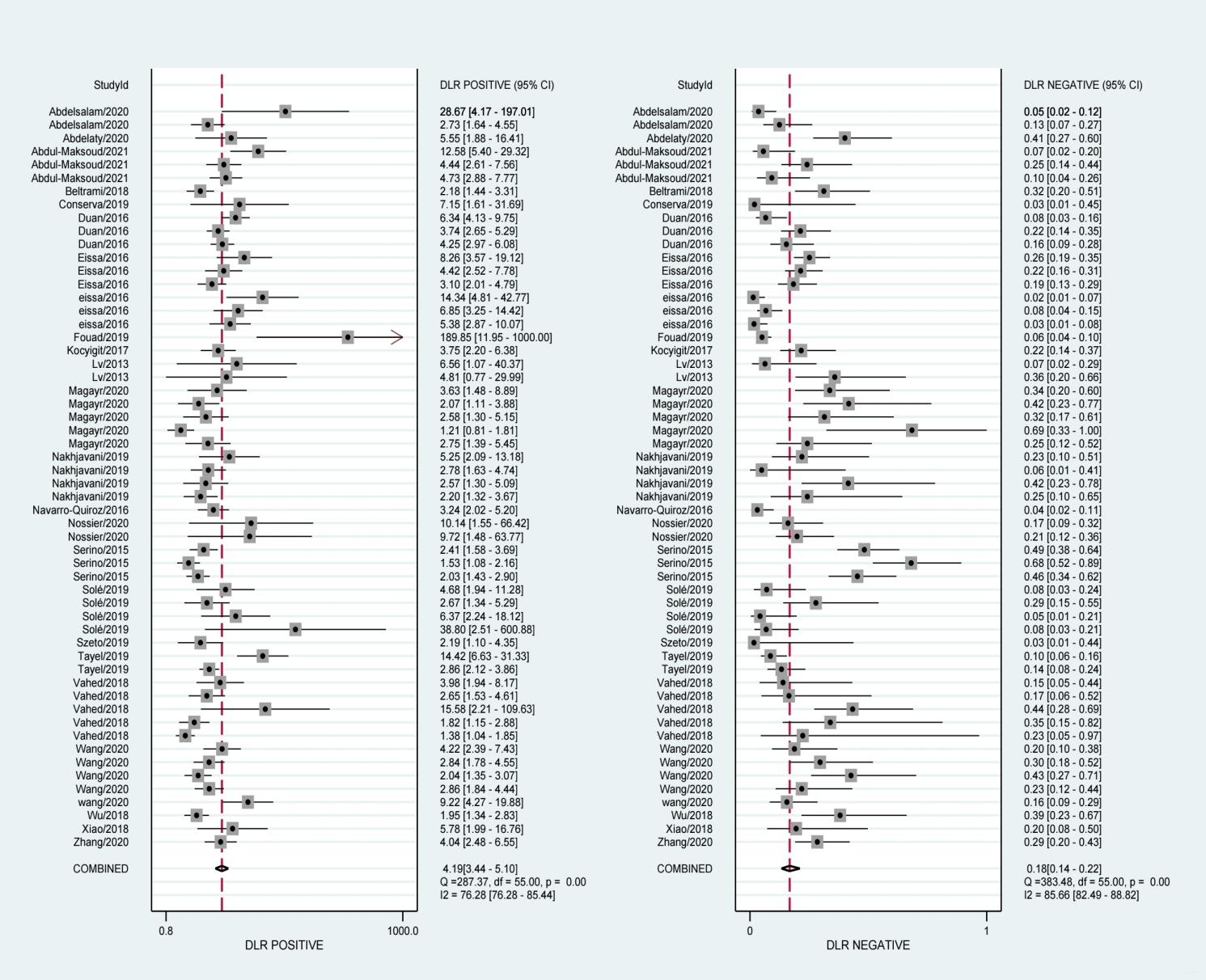


S5 Forest plots of positive and negative likelihood ratios


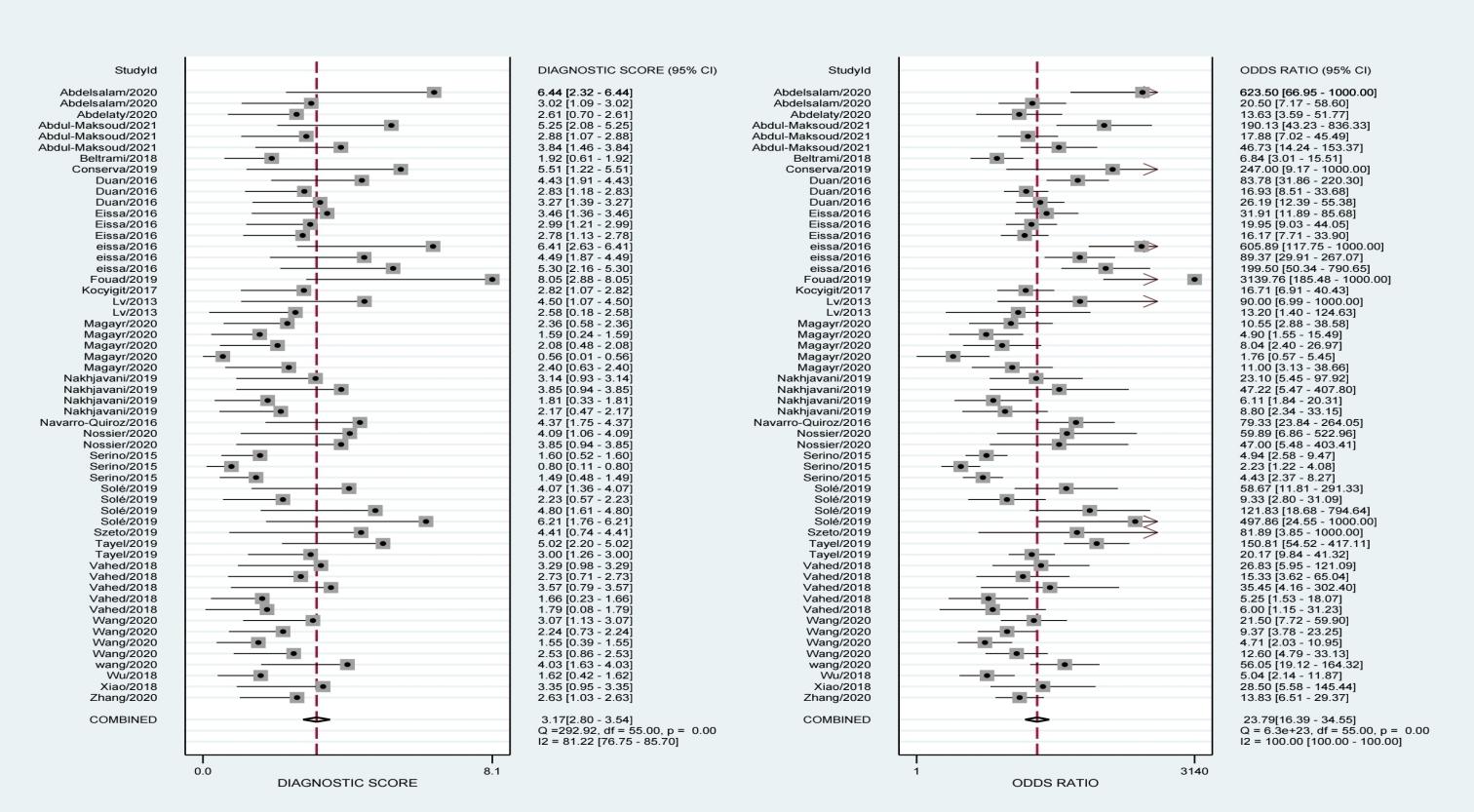


S6 Forest plots of diagnostic odds ratios


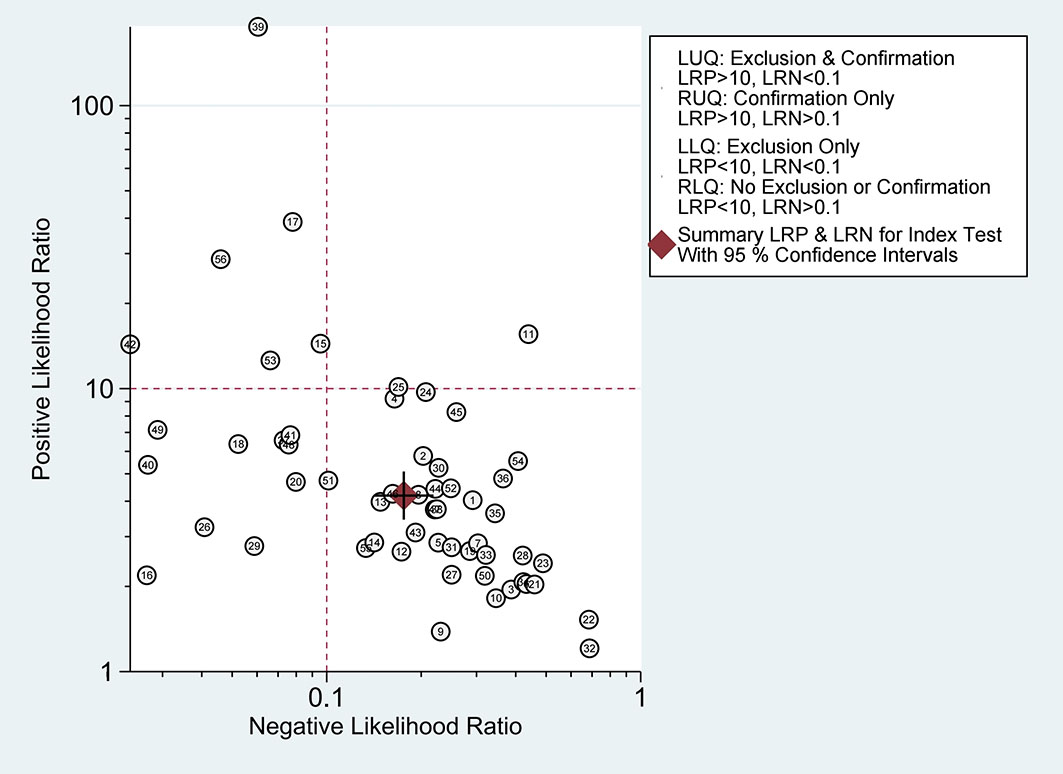


S7 Summary of positive likelihood ratio and

negative likelihood ratio for diagnosis of CKD
